# Enteropathic SAPHO Syndrome in Ulcerative Colitis Responsive to Bisphosphonates

**DOI:** 10.1155/2024/3558853

**Published:** 2024-10-16

**Authors:** Jordan Phillipps, Sehreen Mumtaz, Jayesh Valecha, Rupert O. Stanborough, Florentina Berianu, Ejigayehu Abate, Vikas Majithia

**Affiliations:** ^1^Division of Rheumatology, Mayo Clinic, Jacksonville 32224, Florida, USA; ^2^Division of Radiology, Mayo Clinic, Jacksonville 32224, Florida, USA; ^3^Division of Endocrinology, Mayo Clinic, Jacksonville 32224, Florida, USA

**Keywords:** bisphosphonates, case report, Crohn's disease, enteropathic SAPHO syndrome, inflammatory bowel disease, SAPHO (synovitis, acne, pustulosis, hyperostosis, osteitis) syndrome, ulcerative colitis

## Abstract

SAPHO syndrome, a rare inflammatory disorder of bone, joints, and skin, is named based on the presence of synovitis, acne, pustulosis, hyperostosis, and osteitis. The hallmark of SAPHO syndrome includes osteoarticular and dermatologic manifestations, however, rarer associations with inflammatory bowel disease (particularly Crohn's disease) have been documented. The literature on the relationship between SAPHO syndrome and inflammatory bowel disease (IBD), especially ulcerative colitis (UC), remains limited. We report an unusual case of SAPHO syndrome in a patient with UC. Chest x-ray and MRI showed enlargement of the right first rib and adjacent sternum. Bone scintigraphy revealed hyperostosis and ankylosis of the costochondral junction, and bone biopsy revealed reactive bone and costal cartilage without findings of infection or malignancy. Complete resolution of symptoms was achieved 4 months after starting zoledronic acid without significant adverse events. The diagnosis of SAPHO syndrome in IBD patients is rare, even more so in UC patients, likely attributable to underdiagnosis given the clinical heterogeneity of SAPHO syndrome and overlap with the extra-intestinal manifestation of IBD. Our treatment approach provides critical data to the underreported literature on diagnosis and managing SAPHO syndrome in UC.

## 1. Introduction

SAPHO syndrome, a rare inflammatory disorder of bones, joints, and skin that was first described by Chamot in 1987 [1], is named after the presence of synovitis, acne, pustulosis, hyperostosis, and osteitis. SAPHO syndrome pathogenesis is not well understood, but it is commonly described as an autoinflammatory disorder with genetic, environmental, and immune components contributing to the disease process. SAPHO syndrome is a rare disease, with an estimated prevalence of 0.01%, and typically affects young-to-middle-aged adults (between 30 and 50 years with a female predominance); however, it can present at any age [2–4]. The hallmark of SAPHO syndrome includes osteoarticular and dermatologic manifestations; however, there are reports of a rare association with inflammatory bowel disease (IBD), particularly Crohn's disease [5]. The literature on the relationship between SAPHO syndrome and IBD, especially ulcerative colitis (UC), and subsequent disease management remains limited, warranting further research to address such gaps. Here, we report a unique case of SAPHO syndrome in a patient with UC that was successfully treated with local steroid injections and zoledronic acid.

## 2. Case Summary

**Figure 1:**
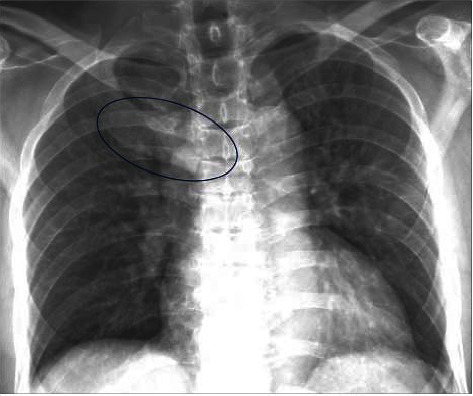
Posterior–anterior chest radiograph showing enlargement of the anterior right first rib and chondral cartilage with cortical thickening.

**Figure 2:**
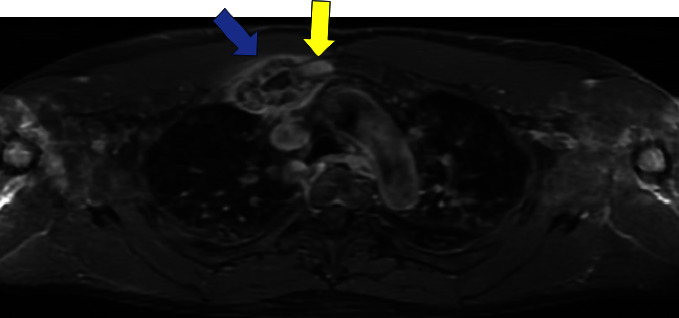
Axial T1-weighted fat-saturated postcontrast MRI of the upper thorax showing enlargement of the right first rib (blue arrow) at the costochondral junction involving the costomanubrial articulation. The chondral rib is enlarged with a thick osseous rim that intrinsically enhances. The adjacent marrow of the manubrium and surrounding soft tissues enhances (yellow arrow).

**Figure 3:**
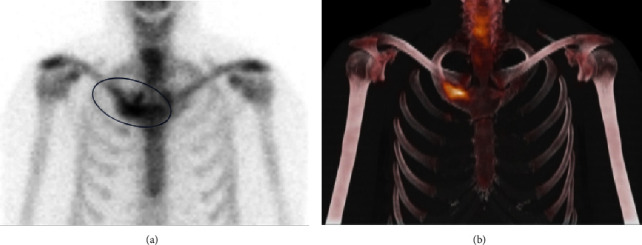
Bone scan with SPECT/CT imaging following technetium-99 m medronate injection (Tc-99 m MDP). Planar projection of the thorax (a) and 3D maximum intensity projection (MIP) CT + SPECT (b) show increased radiotracer uptake in the right first rib and costochondral junction corresponding with the enlarged bone.

**Figure 4:**
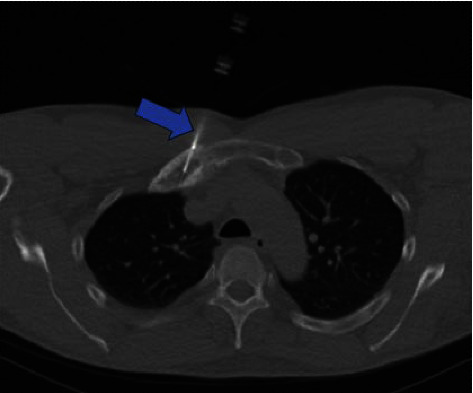
Axial CT-fluoroscopic image of the upper thorax during percutaneous biopsy of the right first rib chondral abnormality using a 13-gauge bone biopsy needle (blue arrow).

A 60-year-old male was referred to our Rheumatology department for further evaluation and management of an unknown autoimmune disorder. He had a 7-year medical history of UC (diagnosed in 2016) treated with various biologic agents, including infliximab (discontinued in June 2019 after 16–18 months due to the development of pustular psoriasis in March 2019), vedolizumab (August 2019 through March 2020; discontinued due to developing pulmonary emboli), and currently ustekinumab (well-controlled on 90 mg every 8 weeks; ongoing since August 2019). He initially presented with a several-year history of persistent right-sided chest pain (beginning May 2019) that was exacerbated by movement and radiated to the right arm and upper back. He had previously trialed NSAIDs with minor relief, and oral glucocorticoids for many months with moderate symptom relief, however, steroid discontinuation caused a flare of his chest pain. He had also previously received a local glucocorticoid injection with adequate relief for several months before presenting to our clinic. He denied any history of inflammatory polyarthritis, warm or swollen joints, constitutional symptoms, or new dermatologic lesions (his previous pustular psoriasis resolved after infliximab discontinuation). Physical examination revealed tenderness of the medial aspect of the right clavicle. Routine laboratory studies revealed elevated inflammatory markers (erythrocyte sedimentation rate [ESR] 92 mm/hr, C-reactive protein [CRP] 25 mg/L), while complete blood counts, an autoimmune panel (including Scl-70, rheumatoid factor, double-stranded DNA, anti-SSA/SSB, c-ANCA, p-ANCA, ANA, RNP, RNA polymerase III, and Jo1) and liver function tests were within normal limits. HLA-B27 testing was negative. Radiographic assessment, including a chest X-ray (Figure 1) and magnetic resonance imaging (MRI) (Figure 2), showed enlargement of the right first rib and adjacent sternum. Whole-body bone scintigraphy (Figure 3) showed an expansile lesion in the distal right first rib with associated hyperostosis and ankylosis of the costochondral junction, without any other axial and/or appendicular skeleton findings. A bone biopsy (Figure 4) of the right anterior first rib revealed reactive bone and costal cartilage without findings of infection or malignancy. Given the absence of other competing etiologies, a diagnosis of SAPHO syndrome was made (in 2023). He was started on treatment with sulfasalazine (2 g daily) with local glucocorticoid injections (every 4–6 weeks). Notably, other anti-TNFs were deferred given his prior history of infliximab-induced pustular psoriasis, and methotrexate was deferred given his reluctance to reduce frequent social drinking. This approach provided moderate symptom relief; however, he experienced significant bloating while on sulfasalazine, prompting its discontinuation 1 month later. He was subsequently started on zoledronic acid (1g) 4 months later with complete symptom resolution during a 4-month follow-up.

## 3. Discussion

The present study reports a unique case of osteoarticular SAPHO syndrome in a patient with ulcerative colitis treated with local steroid injections and bisphosphonates. The patient tolerated treatment well without significant adverse events and exhibited a great clinical response at 9 months of follow-up.

SAPHO syndrome exhibits clinical heterogeneity and lacks validated diagnostic criteria, which poses significant diagnostic challenges for clinicians. SAPHO syndrome is often a diagnosis of exclusion, requiring a thorough history, physical examination, and radiographic/laboratory assessments to rule out alternative etiologies, such as malignancy, osteomyelitis, and seronegative spondyloarthropathies. Osteoarticular manifestations, most notably hyperostosis and/or osteitis, are a hallmark of SAPHO syndrome, irrespective of active dermatologic findings [2, 6]. Musculoskeletal changes are typically identified on plain radiographs and advanced imaging (e.g., CT, MRI, and bone scintigraphy) [7, 8]. Laboratory findings often include elevations in nonspecific inflammatory markers, including ESR and CRP. Our patient's current presentation seemingly lacked cutaneous manifestations of SAPHO syndrome (e.g., acneiform [commonly nodulocystic acne] and neutrophilic [commonly palmoplantar pustulosis] dermatoses), but the presence of isolated osteoarticular involvement (right sternoclavicular joint hyperostosis) in the context of ulcerative colitis and absence of alternative etiologies (no evidence of malignancy or osteomyelitis on bone biopsy; no evidence of sacroiliac joint involvement on imaging and HLA-B27 negative), made SAPHO syndrome the most likely diagnosis [9]. Our patient had a recent history of pustular psoriasis attributable to infliximab (supported by symptom resolution upon infliximab discontinuation); however, the pustular skin lesions of SAPHO syndrome and pustular psoriasis are clinically indistinguishable, making it difficult to completely rule out dermatologic manifestations of SAPHO syndrome in the present case. Notably, skin lesions are absent in approximately one-third of SAPHO syndrome patients, and most dermatologic manifestations occur within 2 years of diagnosis, suggesting that there are unknown, underlying factors predisposing to dermatologic manifestations in SAPHO syndrome [10].

There are no standardized treatment algorithms for SAPHO syndrome due to its rarity and consequent paucity of available literature; current treatment approaches are based on observational data. Osteoarticular manifestations (without dermatologic components) are initially treated with NSAIDs, followed by short courses of oral glucocorticoids [11]. Steroid-sparing immunosuppressive agents (e.g., methotrexate and tumor necrosis factor [TNF]-alpha inhibition) are used in refractory cases (either due to inadequate response or intolerance of initial agents). For patients with cutaneous manifestations, oral tetracyclines and oral retinoids may be beneficial [12]. IL-17 and IL-12/23 inhibition, sulfasalazine, Janus kinase (JAK) inhibition, and bisphosphonates, have reported efficacy in treating refractory cases of osteoarticular disease [13–16]. Relatedly, local intra-articular glucocorticoids have been observed to provide significant symptom relief for SAPHO syndrome patients with sternocostoclavicular joint involvement (which was also true for our patient with right sternoclavicular involvement) [17]. Our patient had minimal relief with NSAIDs, moderate relief from intermittent glucocorticoid injections and oral corticosteroids, and a positive response to escalation of care with sulfasalazine (which was discontinued due to intolerance) and zoledronic acid. This supports the use of disease-modifying antirheumatic drugs (DMARDs) and bisphosphonates in treating SAPHO syndrome in patients with ulcerative colitis. Of note, our patient was concurrently taking ustekinumab (anti-IL-12/23) for his ulcerative colitis, which has been reported to be effective in SAPHO syndrome [13]. However, he had been on ustekinumab for several months at the time of his presentation to our clinic (while actively symptomatic), suggesting that it was not controlling his osteoarticular symptoms as monotherapy, but might have had a synergistic or augmentative effect when combined with bisphosphonates.

SAPHO syndrome has been reported to have rarer disease manifestations, such as IBD. Both SAPHO and IBD are chronic, inflammatory conditions that can affect multiple organs. The relationship between concurrent SAPHO syndrome and IBD, particularly in the practical context of disease management, is understudied and requires further research. Notably, SAPHO syndrome associated with IBD has a reported female predominance and occurs most frequently in patients with Crohn's disease, with prevalence rates varying from less than 1%–10% [2, 5, 6]. A systematic literature review found that 4.8% of SAPHO syndrome patients also had IBD, whereas 0.2% of IBD patients had SAPHO syndrome, suggesting that a diagnosis of SAPHO syndrome confers an increased likelihood for a concurrent IBD diagnosis (as opposed to the reverse) [5]. A separate study identified 15 patients with both SAPHO syndrome and IBD, similarly reporting a predominance of female patients and Crohn's disease [18]. Interestingly, they reported that IBD was diagnosed before SAPHO syndrome in the majority of cases (14/15; 93.3%), often with a time interval of at least 3 years between diagnoses (10/15; 66.7%) [18]. The literature specifically addressing SAPHO syndrome in ulcerative colitis patients remains limited, consisting predominantly of case reports. One case involving a 39-year-old with UC observed a significant clinical response to pamidronate [19], while another case involving a 54-year-old man with UC documented a positive response to tofacitinib (a JAK inhibitor) [14], further suggesting the utility of bisphosphonates and biologics in treating SAPHO syndrome. Ultimately, the diagnosis of SAPHO syndrome in IBD patients is both rare and challenging, with several studies reporting a prevalence of less than 1% [5]. Underdiagnosis may contribute to this rarity, as the clinical features of SAPHO syndrome can mimic the extra-intestinal manifestations of IBD (e.g., neutrophilic dermatoses). Thus, the present study adds crucial clinical context and critical data to the underreported literature on enteropathic SAPHO syndrome in UC with subsequent management approaches.

We report an unusual case of SAPHO syndrome in a patient with ulcerative colitis. The diagnosis of SAPHO syndrome in IBD patients is rare, even more so in ulcerative colitis patients, as most studies have documented a Crohn's disease-predominant IBD association. Underdiagnosis may contribute to this rarity, as SAPHO syndrome displays heterogeneously complex clinical manifestations that can resemble other dermatologic and rheumatic diseases, including the extra-intestinal manifestations of IBD. Ultimately, further investigation of enteropathy SAPHO syndrome variants is warranted to determine the extent of association with IBD (and particularly UC) and to facilitate early recognition of concurrent disease and optimal management and improved outcomes in affected individuals.

## Data Availability

Data sharing is not applicable to this article as no new data were created or analyzed in this study.

## Nomenclature

SAPHO: Synovitis, acne, pustulosis, hyperostosis, osteitis syndrome
IBD: Inflammatory bowel disease
UC: Ulcerative colitis
NSAIDs: Nonsteroidal anti-inflammatory drugs
ESR: Erythrocyte sedimentation rate
CRP: C-reactive protein
c-ANCA: Cytoplasmic antineutrophil cytoplasmic antibody
p-ANCA: Perinuclear antineutrophil cytoplasmic antibodies
RNP: Ribonucleoprotein
ANA: Antinuclear antibody
MRI: Magnetic resonance imaging
TNF: Tumor necrosis factor
IL: Interleukin
JAK: Janus kinase
DMARDs: Disease-modifying antirheumatic drugs
HLA: Human leukocyte antigen.
