# Correction to “Enteropathic SAPHO Syndrome in Ulcerative Colitis Responsive to Bisphosphonates”

**DOI:** 10.1155/crrh/9860969

**Published:** 2025-07-09

J. Phillipps, S. Mumtaz, J. Valecha, et al., “Enteropathic SAPHO Syndrome in Ulcerative Colitis Responsive to Bisphosphonates,” *Case Reports in Rheumatology* 2024 (2024): 3558853, https://doi.org/10.1155/2024/3558853.

In the article, there is an error in the stated dosage of zoledronic acid given to the patient. The correct dosage is 5 milligrams of zoledronic acid.

The authors apologize for this error.

